# Gut Bacteria in the Holometabola: A Review of Obligate and Facultative Symbionts

**DOI:** 10.1093/jisesa/ieaa084

**Published:** 2020-08-20

**Authors:** R A Kucuk

**Affiliations:** Clemson University, Poole Agricultural Center, Clemson, SC

**Keywords:** nutrition, microbiome, endosymbionts, transmission, gut

## Abstract

The diversity and ecological variety of Holometabola foregrounds a wide array of dynamic symbiotic relationships with gut-dwelling bacteria. A review of the literature highlights that holometabolous insects rely on both obligate bacteria and facultative bacteria living in their guts to satisfy a number of physiological needs. The driving forces behind these differing relationships can be hypothesized through the scrutiny of bacterial associations with host gut morphology, and transmission of bacteria within a given host taxon. Our knowledge of the evolution of facultative or obligate symbiotic bacteria in holometabolan systems is further enhanced by an assessment of the various services the bacteria provide, including nutrition, immune system health, and development. The diversity of Holometabola can thus be examined through an assessment of known bacterial partnerships within the orders of Holometabola.

Many microorganisms colonize the hyperdiverse Holometabola (insects that exhibit complete metamorphosis), including bacteria inhabiting the digestive system (Dillon et al. 2004). Some of these bacteria are transient commensals (Zhang et al. 2016, Hammer et al. 2017), whereas others are capable of effectively colonizing and reproducing in a host and attending to physiological needs for a portion of its lifespan (Salem et al. 2017). Various symbionts can be found across this spectrum, and they can be classified as obligate (host-restricted) or facultative (nonhost-restricted) (Fisher et al. 2017). In the Holometabola, obligate gut bacteria have effectively integrated themselves morphologically or physiologically with the host, to the point of mutualistic interdependence. Yet, various facultative bacteria also provide key services to the host without being beholden to it for survival, or necessarily receiving reciprocation for their services.

All major insect orders have representatives that possess a microbiome (Kibuchi 2009), but the holometabolans show a striking taxonomic diversity and ecological variety that may permit a greater diversity of bacterial partnerships. The novel physiological process of complete metamorphosis, involving distinctive larval, pupal, and adult stages, has been implicated in this phylogenetic and ecological diversity in Holometabola (Yang 2001, Rainford et al. 2014), as well as the variety of bacterial symbioses in holometabolan guts (Hammer and Moran 2019). Given the potential of symbiosis presented by this immense multipartite variation, the biodiversity of holometabolous insects can be seen as a particularly broad window into the numerous dynamic relationships between host and gut symbiont. Despite gaps in knowledge, meaningful understanding of the evolution of these relationships can begin by highlighting their presence in the holometabolan tree of life.

We also can shed light on the evolution of bacterial symbiosis in the digestive tract of Holometabola by exploring the particular ways bacteria are situated in the gut, how these bacteria are transmitted between hosts (including different developmental stages), and how they assist in major physiological needs of the host. From there, we can further elucidate the diversity of the Holometabola through identification of gut bacterial symbioses in the context of host clade. Finally, we can ask how these relationships may have arisen. The purpose of this review is to synthesize current knowledge on gut-dwelling symbiotic bacteria in the Holometabola through an examination of their presence in the context of transmission, location of settlement, services provided, and host taxonomy. This review aims ultimately to demonstrate the need to fill in the enormous gaps in knowledge of the functional biology and evolution of symbioses centered around the digestive tract of Holometabola and also address the areas in which data is lacking.

## Host Gut Tissue and Morphology

How a type of bacteria colonizes a gut in various physiological contexts is partly related to the tissue and morphology of the digestive system (Donaldson et al. 2016). The diversity of bacteria as well as the specifics of their relationship with the host are often dependent on gut structure (Donaldson et al. 2015). The holometabolan gut shows numerous adaptations to facilitate certain diets, including morphologically distinct and often subdivided foregut, midgut, and hindgut regions in addition to the presence of peritrophic matrices (Engel and Moran 2013). Further morphological modification has allowed for bacterial settlement and storage: this includes specialized cells called bacteriocytes, diverticula and crypts known as bacteriomes, and wholesale modifications to the regions of the gut (Engel and Moran 2013, Ceja-Navarro et al. 2019). While the strategies of bacterial gut habitation are varied and frequently overlap, facultative and obligate gut bacteria can be placed in two basic categories: endocellular symbionts and free-living symbionts. Both of these types are either associated with special storage sites or localization in particular regions of the gut (Engel et al. 2013).

Symbiotic gut bacteria can inhabit particular cells of the digestive system of their hosts. This situation is common in the midgut epithelium, as exhibited by obligate bacteria in carpenter ants (*Camponotus*) (Mayr, Hymenoptera: Formicidae) (Schröder et al. 1996, Ratzka et al. 2013), tsetse flies (*Glossina* spp.) (Weidemann, Diptera: Glossinidae) (Wang et al. 2013), and larval olive flies (*Bactrocera oleae*) (Rossi, Diptera: Tephritidae) (Estes et al. 2009, Ben-Yosef et al. 2010). Foregut intracellular bacteria are known as well, as seen in the bacteriome-inhabiting obligate *Nardonella* of some weevils (Buchner 1965, Anbutsu et al. 2017). The gut region and cell type a symbiont settles in can potentially inform us about the particulars of host physiology and symbiont service, but they might also tell us about how such intimate associations can evolve. The presence of obligates that switch between intracellular and extracellular habitation after metamorphosis (Estes et al. 2009) demonstrates a flexibility that may inform us about the development of gut symbiosis in Holometabola. We can also question how such relationships arise in the Holometabola by examining how long-standing obligate symbionts (Toju et al. 2013, Williams and Wernegreen 2015), can be ousted by new obligate symbionts, as seen in some true weevils (Toju et al. 2013).

Localization of bacterial symbionts in the gut also occurs extracellularly in bacteriomes (Engel and Moran 2013). The leaf beetles *Cassida rubiginosa* (Müller, Coleoptera, Chrysomelidae) and *Bromius obscurus* possess clear obligate-housing bacteriome regions around the foregut-midgut junction (Stammer 1936, Fukumori et al. 2017, Salem et al. 2017). The presence of bacteriomes in certain gut regions and the presence of free-living bacteria in these structures raises an important question about the how holometabolan hosts may facilitate beneficial bacteria in their guts: which evolves first, the bacteriome or the bacteriocyte? The fact that both endocellular symbionts and extracellular symbionts inhabit bacteriomes might help us arrange a picture of a hypothetical ‘intermediate’ condition prior to heightened integration. The presence of strong immune responses to endocellular symbionts experimentally introduced to areas outside of their bacteriomes, as seen in a weevil (*Sitophilus zeamais*) (Anselme et al. 2008) suggests that we must also consider certain physiological constraints.

Broader modifications of the gut can be associated with distinctive, localized bacterial communities—a phenomenon of gut compartmentalization (Engel and Moran 2013). This is observed in Holometabola with hindguts that perform putative storage functions, like scarab beetles and their relatives, whose midguts and hindguts harbor their own distinctive communities (Egert et al. 2003, Andert et al. 2010, Zhang et al. 2018 and whose hindgut bacteria are considered to be beneficial (Huang et al. 2012, Ceja-Navarro et al. 2014). A heavily modified paunchlike hindgut harboring beneficial bacteria is found in the larvae of the crane fly *Tipula abdominalis* (Say, Diptera: Tipulidae) as well (Cook et al. 2007) . The preference for certain regions of the digestive system by symbiotic bacteria is not restricted to the hindgut. For example, adult vinegar flies maintain a community of environmentally acquired symbionts of the genus *Acetobacter* (Beijernek, Rhodospirallales: Acetobacteraceae) in the foregut crop (Pais et al. 2018, Ma and Leulier 2018), and olive flies have bacteria-foregut associations (Ben-Yosef et al. 2014). How the host ensures that important organisms remain ‘at their stations’ in a tumultuous flow-through gut without inhabiting a bacteriocyte or bacteriome requires a closer examination of these distinctive structures. In the ant *Cephalotes rohweri* (Wheeler, Hymenoptera: Formicidae), for example, a foregut filter inhibits the spread and growth of certain bacteria (Lanan et al. 2016) and in vinegar flies (*Drosophila melanogaster*) (Meigen, Diptera: Drosophilidae), a diverticula-like crop provides a site of bacterial proliferation (Pais et al. 2018). Analysis of such morphological compartmentalization in addition to surrounding physiological constraints (like the immune system) may help explain where bacteria settle, and ultimately where they become more permanent fixtures of the gut.

## Mechanisms of Transmission

Holometabolous insects demonstrate both vertical (mother-to-offspring) and horizontal (environment-to-host) transmission of bacterial gut symbionts (Hammer and Moran 2019). However, a metamorphic period marked by an expulsion of gut contents and a modification of gut morphology from larval to pupal and adult stages can potentially interfere with bacterial symbiont colonization (Moll et al. 2001, Engel and Moran 2013). Thus, holometabolous insects are faced with the challenge of passing on beneficial bacteria to the next generation as well as ensuring that such bacteria are retained into the adult stage. Within the basic schema of vertical and horizontal transmission strategies, Holometabola and their bacterial symbionts demonstrate several ways to overcome inter- and intra-generational hurdles.

Some Holometabola have guaranteed transmission of symbiotic bacteria through vertical transmission, and known obligate bacteria are transmitted exclusively through this route (Douglas 2015). For example, the female thistle tortoise beetle *C. rubiginosa* possesses specialized atria in the reproductive tract that harbor the obligate symbiotic bacteria *Stammera* so it can be deposited on eggs and bestowed to hatching larvae in the form of a protected caplet (Salem et al. 2017). Gut symbionts of Holometabola also vertically make it to the next generation via association with the female oocytes, as seen in weevils with the obligate symbionts *Nardonella* and *Sodalis* (Enterobacteriales: Pectobacteraceae) (Anbutsu et al. 2017, Zaidman-Rémy et al. 2018). Vertical transmission of obligate symbionts can be accomplished via glands, as in the milk glands of female tsetse flies (*Glossina*) (Zaidman-Rémy et al. 2018). There is vertical transmission of facultative bacteria as well: in the burying beetle *Nicrophorus vespilloides* (Herbst, Coleoptera: Silphidae), bacteria in the genus *Dysgonomonas* (Hostad et al., Bacteroidales: Porphyromonadaceae) are transmitted to the next generation via gut (oral and anal) secretions on food substrate (Wang et al. 2017). Some dung beetle mothers are capable of providing their larvae with facultative bacterial inoculates in the form of a microorganism-rich brood ball or pedestal (Estes et al. 2013, Schwab et al. 2016, Shukla et al. 2016). Given that obligate symbionts are invariably transmitted through host without exposure to the external environment (Douglas 2015) the development of their vertical transmission is a key evolutionary question. Some vertically transmitted nongut symbionts in Holometabola do not provide services for adults, as we see in the *Burkholderia* (Yabuuchi et al., Burkholderiales: Burkholderiaceae) of darkling beetles (Kaltenpoth and Flórez 2020), and whether or not concerted gut associated symbiosis must exist before reproductive-organ-associated vertical transmission occurs should be explored further.

Inter- and intra-generational transmission of beneficial gut bacteria can be solved purely through horizontal means. Gut bacterial taxa in general can be shared between insects and their surrounding environments (Ziganshina et al. 2018), and this is also the case for holometabolans harboring symbiotic bacteria. For example, beneficial bacteria that are only necessary during a single life stage, such as the facultative gut bacteria of larval mosquitos (*Aedes aegypti*) (Linnaeus, Diptera: Culicidae), are ingested by their hosts through feeding in the surrounding environment (Coon et al. 2016, 2017, 2020). Shared adult and larval habitat can act as multigenerational bacterial reservoirs for horizontal transmission, as in the coffee berry borer *Hypothenemus hampei* (Ferrari, Coleoptera: Curculionidae) (Mariño et al. 2018) and its facultative bacteria. The acquisition of symbiotic facultative bacteria through food is demonstrated in vinegar flies as well, through the defecation and regurgitation of hosts into food sources and subsequent re-acquisition (Pais et al. 2018, Storelli et al. 2018). The ‘environmental reservoir’ solution to ensuring horizontal transmission of bacteria is also observed in eusocial Holometabola, like the honey bee *Apis mellifera* (Linnaeus, Hymenoptera: Apidae) whose siblings ensure transmission of beneficial microbes via trophallaxis (Lanan et al. 2016)—a colony wide ‘social stomach’ (Tarpy et al. 2015, Kwong and Moran 2016). This situation may explain the general trend in social insect symbionts like *Gilliamella* (Kwong and Moran, Orbales, Orbaceae) that are fairly restricted to a specific host on the generic level (Douglas 2015) despite being capable of living outside of these hosts (Zheng et al. 2016). Such environments may foster a relationship between host and symbiont that leads to integration and may also prevent the need for further integration if the required symbionts are present in the food source that can be readily ingested.

## Host Nutrition

Diet alone affects the presence and diversity of resident bacteria (Yun et al. 2014, Kim et al. 2017, Kudo et al. 2019). A wide array of holometabolan diets demonstrate food-gut bacteria confluence, from nectar-feeding, as seen in certain adult Lepidoptera (Phalnikar et al. 2018), to wood-feeding, as seen in some adult and larval Coleoptera (Berasategui et al. 2016). A food source can be more generally associated with a particular bacterial community regardless of the identity of the host feeding on it: conifer-bark-feeding weevils (*Hylobius abietis*) (Linnaeus, Coleoptera: Curculionidae) harbor a distinctive bacterial community that is both conserved across populations and bears a general similarity to the gut community of other beetles feeding on the same diet (Berasategui et al. 2016). Nutritional association is not purely commensal, however, and holometabolous gut bacteria are implicated in nutritional symbioses centered around food breakdown, nutrient provisioning, and detoxification.

An important way in which bacteria can assist in nutrition is through digestion of food material (Brune 2009). In Holometabola, there is a relationship between diets rich in structurally recalcitrant food material and gut bacteria with the metabolic tools to break it down. For example, foliovores like moth and butterfly larvae harbor facultative bacteria capable of digesting cellulose and xylan in leaves (Pinto-Tomás et al. 2007, Anand et al. 2010) and pectin and starch digestion in caterpillars is assisted by facultatives as well, as in the silkworm moth caterpillar *Bombyx mori* (Linnaeus, Lepidoptera: Bombycidae) (Anand et al. 2010). Xylophagy in the holometabolans is associated with facultative digestive bacteria—longhorn beetle larvae house gut bacteria with genes associated with lignocellulose breakdown (Mohammed et al. 2018) and adult and larval bark and turpentine beetles (*Dendroctonus* spp.) (Erichson, Coleoptera: Curculionidae) harbor cellulolytic gut bacteria (Morales-Jiménez et al. 2012, Hu et al. 2019), as do wood wasps (*Sirex noctilio*) (Fabricius, Hymenoptera: Siricidae) (Adams et al. 2011). Facultative digestive bacteria are present in rhizophages, such as the cellulolytic taxa of the scarab *Holotrichia parallela* (Motschulsky, Coleoptera: Scarabaeidae) (Huang et al. 2012). Holometabolans feeding on heavily degraded plant material, like herbivore dung and humus also exhibit facultative digestive communities, as we see in larval dung beetles (*Onthophagus taurus*) (Schreber, Coleoptera: Scarabaeidae) (Estes et al. 2013) and larval flower chafers (*Pachnoda marginata*) (Drury, Coleoptera: Scarabaeidae) (Cazemier et al. 2003). The majority of known bacteria exhibiting these digestive activities in insects are facultative, although there are exceptions of obligate mutualists fulfilling this role, as in the pectin-degrading bacteria of foliovorous tortoise beetles (*C. rubiginosa*) (Salem et al. 2017). Given that some lineages of facultative digestive bacteria do get passed on to their offspring (Estes et al. 2013), the possibility of host-specific (i.e., more integrated) lineages of environmental bacteria providing a testable model for symbiosis development is promising. In scrutinizing the preponderance of facultative digestive bacteria in holometabolan guts, we must also consider the implications host morphology and routes of transmission have on this service and the symbionts that provide it.

Gut-associated bacteria can be utilized to make up for the low nutritional quality of a food source and symbiotic bacteria are found in hosts whose diets are nutritionally impoverished (Buchner 1965). For example, bacteria can perform nitrogen fixation in insects with carbohydrate-rich diets, as in the facultative bacteria of pollenivorous ants, wood-feeding longhorn beetles, bark beetles and bess beetles, and in root-feeding weevils (Morales-Jiménez et al. 2009, Russell et al. 2009, Reid et al. 2011, Morales-Jiménez et al. 2013, Shelef et al. 2013, Ceja-Navarro et al. 2014, Ayayee et al. 2016). Adult pine weevils feeding on pine wood benefit from facultative gut bacteria that metabolize diterpenes in their food substrate to enhance its nutritional value (Berasategui et al. 2017). Obligate symbionts are associated with nutrient provisioning as well, as demonstrated by the tsetse fly (*Glossina* spp.) bacteria *Wigglesworthia* (Aksoy, Enterobacteriales: Erwiniaceae), which aids in the synthesis of B vitamins (Wang et al. 2013, Griffith et al. 2018) and the olive fly gut bacteria that assist in amino-acid provisioning to supplement the honeydew diet of their hosts (Ben-Yosef et al. 2010). Obligate nutrient provisioners are directly implicated in developmental success, as in *Nardonella* and *Sodalis*, which synthesize tyrosine that aids in durable exoskeleton construction in the herbivorous weevils *Pachyrhynchus infernalis* (Fairmaire, Coleoptera: Curculionidae) and *Sitophilus* spp., respectively (Vigneron et al. 2014, Anbutsu et al. 2017). Additionally, why some nutrient provisioners are obligates instead of facultatives can potentially tell us about the foundation of gut-centric symbiosis versus more ‘promiscuous’ nongut-restricted forms.

Gut bacteria assist in management and suppression of potentially dangerous toxins in and around the host food source. Detoxification can be demonstrated by symbionts exposed to manmade toxins, as seen in the facultative symbionts of cowpea beetles (*Callosobruchus maculatus*) (Fabricius, Coleoptera: Chrysomelidae) exposed to dichlorvos (Akami et al. 2019), and diamondback moths (*Plutella xystostella*) (Linnaeus, Lepidoptera: Plutellidae) exposed to chlorpyrifos (Xia et al. 2018). Bacteria can also assist in processing of food-source-derived toxins, as in the coffee berry borer whose facultative gut bacteria assist in the breakdown of the harmful alkaloid caffeine (Ceja-Navarro et al. 2015) and the facultative gut symbionts of honeybees that break down toxic sugars (Zheng et al. 2016). The presence of facultative organisms in toxin breakdown may be due to the errant nature of the symbionts—environmentally acquired facultatives are not inherently dependent on host metabolism to defend themselves from toxins, natural or manmade. Consequently, relationships with bacteria originating from the environment potentially dominate certain services by virtue of exaptation—what is good for the bacteria is incidentally good for the insect host. The wide range of facultative symbionts assisting in detoxification may be a testament to this—even nonholometabolans that do not face the challenges of maintaining obligates still make use of bacteria for these services (Werren et al. 2012).

Not all diets are challenging, but nutritionally associated symbiotic bacteria abound in the guts of Holometabola dealing with more balanced food sources as well, such as other insects (Zientz et al. 2006, Werren et al. 2012). These include obligate nutrient-provisioning symbionts, like the *Blochmannia* of omnivorous carpenter ants (Zientz et al. 2006). Additionally, predatory Holometabola can harbor the key symbionts of their prey as is the case in the Asian giant hornet (*Vespa mandarinia*) (Smith, Hymenoptera: Vespidae) and Japanese hornet (*V. simillima*) (Smith, Hymenoptera: Vespidae) (Suenami et al. 2019) that hunt honeybees. Whether or not nutritional symbionts are present, and whether prey-associated bacteria provide any important nutritive or digestive services to the host is a particularly valuable question when considering highly biodiverse and widespread predatory groups within the Holometabola.

## Host Defense

The Holometabola, like other animals, are at risk of attack by microorganisms, including bacterial and viral pathogens, fungi, and protists (Lacey et al. 2015). While insects possess an immune system, bacteria can provide assistance in regard to defending their hosts against pathogens and parasites (Brownlie and Johnson 2009). Various forms of pathogen assistance are exhibited by gut bacteria in the Holometabola, and this defensive role can be roughly split into two categories: immune system management and direct defense. Both obligate and facultative bacteria can fill these respective roles in Holometabola (Tables 1–4).

The very development of the immune system is enhanced by gut bacteria (Tables 1–4). Facultative bacteria aid in immune system homeostasis in fruit flies and honeybees (Ryu et al. 2008, Kwong et al. 2017), and in immune system priming in the red palm weevil (Muhammad et al. 2019). Infestations by parasitic eukaryotes are reduced by symbiont-mediated immune priming: trypanosomatids are combated in bumblebees (*Bombus*) (Latreille, Hymenoptera: Apidae) harboring core facultative symbionts (Koch and Schmid-Hempel 2011), and in mosquitoes, the infection rate of the malaria parasite *Plasmodium (Marchiafava and Celli, Haemospororida: Plasmodiidae)* is lessened by the presence of a facultative bacterial microbiome (Dong et al. 2009). Gut bacteria facilitate the resistance against prokaryotic infections as well: the toxin-producing biological pesticide *Bacillus thuringiensis* (Berliner, Bacillales: Bacillaceae) is abated in the moth *Spodoptera exigua* (Hübner, Lepidoptera: Noctuidae) (Hernández-Martínez et al. 2010) and other bacterial pathogens are fought off by symbionts in the moth *Galleria mellonella* (Linnaeus, Lepidoptera: Pyralidae) (Jarosz 1979, Johnston and Rolff 2015) and the red flour beetle (Futo et al. 2015). Immune system priming appears to be the province of facultatives, but it is also exhibited by obligates like *Wigglesworthia*, which stimulates normal expression of immunity-related genes and the production of phagocytic hemocytes in tsetse flies (Weiss et al. 2012). How readily this service evolves between facultative bacteria and their hosts might further our understanding of the services that precede the heightened integration we see in obligates.

The holometabolous host’s need to defend itself from pathogens and parasites is associated with other instances of bacteria-mediated aid. Gut bacteria can directly mount biochemical attacks on the microbial aggressors of their hosts, effectively acting as bodyguards (Table 4). Defense against bacteria is demonstrated in burying beetles whose facultative symbionts inhibit the growth of generalist bacteria on the food of their larvae (Shukla et al. 2018, Heise et al. 2019) and stag beetles harbor bacteria-inhibiting *Klebsiella* (Trevisan, Enterobacteriales: Enterobacteriaceae) that is implicated in a similar defensive function (Miyashita et al. 2015). Parasitic eukaryotes may also be directly controlled by gut symbionts. Bacteria associated with the gut of the fungus-harboring wood wasp (*S. noctilio*) may keep the growth of fungi in check via chitinases (Adams et al. 2011), and similar fungal resistance has been suggested by the facultative gut bacteria of the tobacco cutworm (*Spodoptera litura*) (Fabricius, Lepidoptera: Noctuidae) (Subhashini 2015). Other eukaryotes like nematodes can be combated by gut bacteria, as is the case with the symbionts of burying beetles (Heise et al. 2019). The advantage of facultative organisms versus obligates may be similar to the benefits suggested for detoxification: bacteria that must exist without the protection of an insect immune system may be more versatile in their capacity to fight off potentially harmful pathogens. Moreover, excreted bacterial bodyguards that protect insects from the outside (e.g., on eggs or a larval food substrate) must necessarily be able to function independently of their host to perform effectively.

## Host Lineage

The diversity of Holometabola has been explained in the context of adaptive radiation on flowering plants (Farrell 1998), radiation through parasitism (Forbes et al. 2018), and eusocial behavior (Legendre and Condamine 2018) as well as the key innovation of the group—complete metamorphosis. Among the holometabolan lineages, it is possible key innovations have driven partnership with gut bacteria (Moran et al. 2019). Comparing the four largest holometabolan orders—the Coleoptera-Diptera, Hymenoptera, and Lepidoptera (Condamine et al. 2016)—in the context of the previously mentioned services provided by bacteria, we find examples of obligate symbionts in all but the Lepidoptera (Table 2) while facultatives are distributed in these four orders (Tables 1–4).

Hymenoptera generally harbor gut communities low in taxon richness (Colman et al. 2012) and comprise the vast majority of eusocial species in the Holometabola (Ratnieks and Helanterä 2009). This eusociality influences the gut bacterial communities of species that exhibit it (Otani et al. 2018), and the many known gut bacterial symbioses in Hymenoptera are represented by social taxa (Table 1). For example, trophallaxis and fecal contact between colony members enables a given species to maintain a stable colony of facultative gut bacteria (Koch and Schmid-Hempel 2011, Sanders et al. 2014, Lanan et al. 2016). As mentioned earlier, social behavior may enable bacteria to be maintained in a host’s digestive system without the presence of certain morphological and physiological measures (e.g., bacteriomes, migration to reproductive organs) that may otherwise be necessary to ensure its transmission to different life stages. Thus, the eusociality in Hymenoptera may have facilitated a general reliance on facultative rather than obligate gut symbionts as seen in the honeybees and other Apidae, in which there is a small core community dominated by the facultative *Snodgrassella* and *Gilliamella* (Martinson et al. 2011, Kwong and Moran 2013). Among the ants (Formicidae), there are facultative core gut species, as seen in the pseudomyrmecine *Tetraponera* (van Borm et al. 2002) and neotropical doryline army ants *Eciton* (Latreille, Hymenoptera: Formicidae), *Labidus*, and *Nomamyrmex* (Borgmeier, Hymenoptera: Formicidae) (Łukasik et al. 2017) as well as myrmicine ants of the tribe Cephalotini (Anderson et al. 2012, Hu et al. 2014). Social Hymenoptera symbionts can assist in the priming of the host immune system against potential pathogens (Kaltenpoth et al. 2014, Kwong et al. 2017) and contribute to development (Raymann and Moran 2018). Honey bee and bumble bee health is associated with the presence of a taxonomically limited but highly conserved community of resident bacteria (Koch and Schmid-Hempel 2011, Raymann and Moran 2018). Given the potential for rapid pathogenesis in social species, social Hymenoptera may be indebted to such organisms as a means to stave off disease. Gut bacteria also show capacity for detoxification and nutrient provisioning in social Hymenoptera (Zheng et al. 2016) and the implications of facultative gut bacteria in maintaining colony health have been noted (Engel et al. 2016). Other social Hymenoptera, such as some predatory Vespidae, possess a limited set of core facultative taxa that may come from their food (Suenami et al. 2019). No symbionts have yet been observed in predatory social Hymenoptera, suggesting that both social behavior and diet contribute to the presence of certain symbioses. Despite the various services facultatives provide to social Hymenoptera, obligate gut bacteria appear as well, as is the case of *Blochmannia* in carpenter ants (Ratzka et al. 2013). Interestingly, among the ants, known obligate bacteria are found in omnivorous species rather than specialists. In regard to the inception of obligate symbiont presence, this pattern suggests that dietary specialization may be subordinate to other selective forces. Among nonsocial bees and wasps, variable and transient communities appear to be the norm (Engel et al. 2016). Even so, some nonsocial Hymenoptera exhibit consistent microbial partnerships as well. In *Nasonia* of the parasitoid family Pteromalidae, the gut microbiome appears intimately linked to phylogeny (Brucker and Bordenstein 2013). Interspecific transmission between closely related taxa in *Nasonia* (Ashmead, Hymenoptera: Pteromalidae) results in greatly decreased fitness compared with intraspecific transmission (van Opstal and Bordenstein 2019). Solitary bees of the genus *Megachile* exhibit the bacteria *Sodalis*, which functions as an obligate developmental and nutritional symbiont in some nonhymenopteran taxa, but whose role in solitary bees has not yet been demonstrated (Rubin et al. 2018). The Hymenoptera also provide a useful basis for symbiont evolution: comparing solitary organisms that are closely related to eusocial or semisocial ones, we may be able to further elucidate the differing methods by which symbiotic gut bacteria are shaped by sociality and vice versa. We may also use this contrast to understand why some symbionts remain facultative but nonetheless demonstrate a degree of integration within host physiology.

**Table 1.** Bacterial symbioses present in the guts of Hymenoptera

Host Species	Host family	Symbionts	Symbiont type	Symbiont locality	Symbiont transmission	Symbiont services	Reference
*Apis melliferra*	Apidae	*Gilliamella apicola, Snodgrassiella* spp.	Facultative	Hindgut Ileum wall	Colony members	Immune system priming, toxin degradation	Martinson et al. 2011, Martinson et al. 2012, Kwong and Moran 2013, Tarpy et al. 2015,Kwong and Moran 2016, Zheng et al. 2016, Kwong et al. 2017
*Bombus* spp.	Apidae	*Gilliamella bombicola*, *Snodgrassiella* spp.	Facultative	Hindgut ileum	Colony members	Immune system priming, toxin degradation	Koch and Schmid-Hempel 2011, Zheng et al. 2016
*Cephalotes* spp.	Formicidae	Unspecified	Facultative	Unspecified	Unspecified	Nitrogen fixation	Lanan et al. 2016, Russell et al. 2009, Martinson et al. 2011
*Camponotus* spp.	Formicidae	*Blochmannia* spp.	Obligate	Midgut bacteriocytes	Ovaries	Nutrient provisioning	Schroeder et al. 1996, Zientz et al. 2006, Ratzka et al. 2013
*Sirex noctilio*	Siricidae	*Streptomyces* spp.	Facultative	Unspecified	Unspecified	Digestion	Adams et al. 2011

In both adult and larval Lepidoptera, the bacterial community is relatively unconserved and environmentally acquired (Hammer et al. 2017, Jones et al. 2019). However, general differences in microbiota have been observed across species among the larvae of butterflies (Lycaenidae, Nymphalidae, Hesperiidae, and Papilionidae) (Phalnikar et al. 2018, Van Schooten et al. 2018, Jones et al. 2019). While there is distinction on the taxonomic level in adult butterflies, taxonomic factors as well as ecological ones are generally subordinate to individual differences (Ravenscraft et al. 2018, Minard et al. 2019). Symbiotic bacteria are present in various moth and butterfly families (Table 2). In the moth families Noctuidae and Pyralidae, the facultative bacterial community has been tied to the nutritional biology of larvae (Anand et al. 2010, Dantur et al. 2015), as well as immune defense (Johnston and Rolff 2015). This apparent absence of a stable microbiome in some Lepidoptera while other species benefit from facultative bacteria in their diet demonstrates the value of versatility in symbionts—relationships can readily arise to provide particular services, and the mere capacity to harbor such bacteria is useful to the host. The dearth of obligate gut bacteria in this enormously successful order may enable us to contrast the Lepidoptera with other orders that occupy similar feeding guilds, such as the Coleoptera, which demonstrate more instances of obligate gut symbionts. Considering that even beetles that feed on the same plant tissues as lepidopterans (e.g., leaves, wood) can be equipped with obligate nutritional assistants, it is reasonable to hypothesize that factors additional to nutritional recalcitrance of the plant are a determinant of such relationships. These may involve particulars of gut morphology and physiology, including the manner in which certain beetles process plant tissue, but further hypothesizing about the ancestral beetle and lepidopteran gut physiology will be needed to test this. Additionally, an understanding of the evolutionary context of this relatively symbiont-free Lepidopteran gut may be enriched by studying the closely related Trichoptera, which display radically different diets and a general physiology that may facilitate symbioses with gut bacteria.

**Table 2.** Bacterial symbioses present in the guts of Lepidoptera

Host Species	Host family	Symbionts	Symbiont type	Symbiont locality	Symbiont transmission	Symbiont services	Reference
*Galleria mellonella*	Pyralidae	*Streptococcus faecalis, Enterecoccus mundii*	Facultative	Unspecified	Unspecified	Immune system priming	Johnston and Rolff 2015
*Spodoptera litura*	Noctuidae	*Serratia* spp.	Facultative	Unspecified	Unspecified	Pathogen defense	Subhashini 2015
*Spodoptera exigua*	Noctuidae	Unspecified	Facultative	Midgut	Unspecified	Detoxification	Hernandez-Martinez et al. 2010
*Plutella xylostella*	Plutellidae	*Enterococcus* spp., *Enterobacter* spp., *Serratia* spp.	Facultative	Unspecified	Food substrate	Detoxification	Xia et al. 2018
*Diatraea saccharalis*	Crambidae	*Klebsiella, Stenotrophomonas, Microbacterium, Bacillus, Enterococcus*	Facultative	Unspecified	Unspecified	Digestion	Dantur et al. 2015
*Bombyx mori*	Bombycidae	*Proteus vulgaris, Klebsiella pneumoniae, Citrobacter freundii, Pseudomonas fluorescens, Erwinia*	Facultative	Unspecified	Unspecified	Digestion	Anand et al. 2010

Among the Diptera, differing diets and natural histories reflect a reliance on a variety of facultative organisms (Table 3). Drosophilid flies such as *D*. *melanogaster* possess a microbiome comprised of horizontally acquired facultative gut symbionts (Leitão-Gonçalves et al. 2017, Gould et al. 2018) including a host-unique facultative gut symbiont, *Acetobacter thailandicus* (Pitiwittayakul, Rhodospirallales: Acetobacteraceae) (Ma and Leulier 2018, Pais et al. 2018). The cactus specialist *D. nigrospiracula* (Patterson and Wheeler, Diptera: Drosophilidae) also demonstrates a host-unique community (Martinson et al. 2017). The crane flies (Tipulidae), which feed on decaying plant material in aquatic environments (Canhoto and Graca 1999), meanwhile may benefit from bacteria-mediated digestion (Cook et al. 2007). A testament to the value of facultative bacteria can be observed in mosquitos for whom environmentally acquired symbionts are essential for molting and pupation, which we see in the mosquitoes (Culicidae) (Coon et al. 2014, 2017). Even so, obligate vertically transmitted bacterial symbionts assist in many interesting evolutionary breakthroughs in the group (Table 3), including hematophagy in Glossinidae (Weiss et al. 2012, Wang et al. 2013). The ecological variety of Diptera offers a potentially rewarding means by which to explore differential use of obligates and facultative organisms, and symbiosis research outside of popular model organisms and pestiferous taxa may yield a greater understanding of the history of symbiosis in the context of the group’s overall success.

**Table 3.** Bacterial symbioses present in the guts of Diptera

Host Species	Host family	Symbionts	Symbiont type	Symbiont locality	Symbiont transmission	Symbiont services	Reference
*Drosophila melanogaster*	Drosophilidae	*Acetobacter thailandicus*, *Lactobacillus plantarum*	Facultative	Foregut diverticulum	Food substrate	Nutrient synthesis, Immune system priming	Ryu et al. 2008, Pais et al. 2018, Storelli et al. 2018
*Bactrocera oleae*	Tephritidae	*Acetobacter tropicalis*, *Erwinia dacicola*	Facultative	Foregut diverticulum (adult), midgut bacteriocytes (larva)	Deposited on eggs	Nutrient synthesis	Estes et al. 2009, Kounatidis et al. 2009, Ben-Yosef et al. 2010
*Aedes aegypti*	Culicidae	Varied	Facultative	Midgut	Environment	Development	Coon et al. 2014, 2016, 2017
*Tipula abdominalis*	Tipulidae	Unspecified	Facultative	Hindgut paunch	Unspecified	Digestion	Cook et al. 2007
*Glossina* spp.	Glossinidae	*Wigglesworthia* spp.*, Sodalis glossinidia*	Obligate	Midgut bacteriocytes	Maternal glandular secretions	Nutrient synthesis, Immune system priming	Weiss et al. 2012, Wang et al. 2013, Griffith et al. 2018, Zaidman-Rémy et al. 2018

Coleoptera may owe some of their diversity to the exploitation of plants as a food source during at least part of their life cycles (Farrell 1998, Janz et al. 2006), and this may partly be explained by bacterial assistance. Indeed, of the insect groups that do possess obligate bacterial symbionts, the primarily plant-associated Phytophaga are the most well-represented (Table 4). The diverse leaf beetles (Chrysomelidae) and weevils (Curculionidae) of Phytophaga have obligate bacterial symbionts in their guts that play a role in nutrition (Anbutsu et al. 2017, Salem et al. 2017). The presence of two instances of co-speciation and three instances of symbiosis with an obligate symbiont (Toju et al. 2013) in weevils suggests a broader necessity for obligate symbionts among the plant-feeding beetles. Further survey and functional assessment of symbionts in successful phytophagous beetle lineages that primarily harbor facultative gut bacteria including the Buprestidae (Vasanthakumar et al. 2008, Bozorov et al. 2019) and Cerambycidae (Ayayee et al. 2014, Ayayee et al. 2016, Mohammed et al. 2018) will be necessary to assess the evolution of dependence on obligate bacteria compared with facultatives. The widespread strategy of harboring cellulolytic bacteria in the Scarabaeoidea whose larvae feed primarily on living and dead plant tissue (Egert et al. 2003, 2005; Arias-Cordero et al. 2012; Ceja-Navarro et al. 2014; Zhang et al. 2018; Chouaia et al. 2019) further emphasizes the need to look more precisely at host biology. Patterns based on general feeding guild do little to elucidate bacterial services beyond gross conjecture. Even diverse beetle groups are neglected in regard to functional analysis of bacterial communities. Carabidae are poorly represented in microbiome research, although they show high gut bacteria diversity within their ranks (Kolasa et al. 2019)—a community that may include symbionts: the presence of omnivory in the group demonstrates a link between diet and the advent of facultative symbionts that aid in digestion (Lundgren and Lehman 2010, Schmid et al. 2014). Whether or not similar trends are present in other largely predatory beetle groups (e.g., Staphylinidae) is unknown, but additional studies may shed further light on carnivory and comparatively scant instances of symbiosis in Holometabola. Like the flies, the particularly great ecological diversity of beetles may be greatly elucidated with an increased effort to identify the benefits conferred by gut bacteria.

**Table 4.** Bacterial symbioses present in the guts of Coleoptera

Host Species	Host family	Symbionts	Symbiont type	Symbiont locality	Symbiont transmission	Symbiont services	Reference
*Holotrichia parallela*	Scarabaeidae	*Pseudomonas* spp., *Cellulosimicrobrium* spp.*, Ochrobacterium* spp.	Facultative	Hindgut	Unspecified	Digestion	Huang et al. 2012
*Pachnoda marginata*	Scarabaeidae	*Promicromonospora pachnodae*	Facultative	Hindgut	Unspecified	Digestion	Cazemier et al. 2003
*Nicrophorus vespilloides*	Silphidae	*Providencia* spp.*, Morganella* spp.*, Vagococcus* spp., *Proteus* spp.*, Koukoulia s*pp.*, Serratia* spp.	Facultative	Unspecified	Food substrate (Parental regurgitants)	Pathogen defense, Food substrate enhancement	Wang and Rosen 2017, Shukla et al. 2018, Heise et al. 2019
*Pachyrhynchus infernalis*	Curculionidae	*Nardonella* spp.	Obligate	Foregut bacteriomes	Ovaries	Nutrient synthesis	Anbutsu et al. 2017
*Hypothenemus hampei*	Curculionidae	*Pseudomonas* spp., *Pantoea* spp.	Facultative	Unspecified	Deposited on eggs	Detoxification	Ceja-Navarro et al. 2015, Mariño et al. 2018
*Cyrtotrachelus buqueti*	Curculionidae	L*actococcus* spp.*, Dysgonomonas* spp.*, Serratia* spp.*, Enterrococcus* spp.	Facultative	Hindgut	Unspecified	Digestion	Luo et al. 2019
*Hylobius abietis*	Curculionidae	*Erwinia* spp.*, Rahnella* spp., *Serratia* spp.	Facultative	Unspecified	Food substrate	Nutrient provisioning	Berasategui et al. 2016, Berasategui et al. 2017
*Anoplophora glabripennis*	Cerambycidae	Unspecified	Facultative	Unspecified	Unspecified	Nutrient provisioning	Ayayee et al. 2014, Ayayee et al. 2016
*Cassidina rubiginosa*	Chrysomelidae	*Stammera* spp.	Obligate	Foregut bacteriomes (Adults & Larvae)	Egg-caplet	Digestion	Salem et al. 2017
*Callosobruchus maculatus*	Chrysomelidae	Unspecified	Facultative	Unspecified	Unspecified	Toxin degradation	Akami et al. 2019
*Tribolium castaneum*	Tenebrionidae	Unspecified	Facultative	Unspecified	Food substrate	Immune system priming	Futo et al. 2016

The absence of entire orders, including Strepsiptera, Mecoptera, Neuroptera, Megaloptera, Raphidioptera, and Trichoptera from extensive functional analysis precludes our ability to make sense of the presence of obligate and facultative bacterial symbionts in Holometabola on a broad phylogenetic scale. Members of these groups display unique innovations that may be have an impact or be impacted by gut bacterial relationships: trichopteran larvae have adopted an almost exclusively aquatic lifestyle (including marine habitats; Riek 1977), and Strepsipera are parasites whose females live almost perennially on host arthropods (McMahon et al. 2011). The groups Neuroptera, Mecoptera, Raphidioptera, and Megaloptera are considered to be relictual (Winterton et al. 2017, Lin et al. 2019) and sister to more diverse clades, a position which may be further elucidated by a comparison of gut bacterial partnership in these groups. Further assessment of these neglected clades must include a wider sampling of host taxa and also address symbiont function.

 It may also be useful to compare these holometabolan taxa with the symbionts of other insect groups, particularly the sister clades within Neoptera. Given that symbionts in these latter groups perform similar functions (e.g., nutrient provisioning, pathogen and pesticide defense), knowledge of their evolution may be enhanced with additional phylogenetic characterization. Such characterization of symbionts via wider sampling may also aid in developing hypotheses about the evolutionary potential of certain bacteria to form partnerships, especially considering the relatedness of symbionts (e.g., *Sodalis* and *Stammera* belong to the family *Enterobacteraceae*, and *Wigglesworthia* and *Erwinia* (Enterobacterales: Erwiniaceae) belong to Erwiniaceae). Moreover, seemingly ‘promiscuous’ symbionts like *Burkholderia* and *Sodalis* may also be useful to phylogenetically characterize for the purpose of examining how much the genus forms novel partnerships with insect hosts within the Holometabola compared with other insect groups (Kaltenpoth and Flórez 2020).

## Concluding Remarks

The diversity of holometabolan bacterial taxa, along with difficulties in experimentally manipulating the bacterial community of the gut of holometabolous insects, continues to challenge our ability to develop a well-structured understanding of bacterial services. This struggle is further deepened by the potential of numerous taxa to influence the ‘performance’ of other members of the community, in addition to the various host-specific factors that shape diversity and functionality of bacteria in their guts. While some bacteria have co-evolved with their hosts (Toju et al. 2013), the extent to which symbiotic bacteria drive insect host evolution and why some insects favor the use of facultative and environmentally acquired over highly specialized obligate ones is still a murky question. Also unknown to us is the breadth of ways originally independent organisms ultimately integrate themselves into host physiology to the extent of becoming obligate mutualists, and the evolution of such integration requires further investigation.

Locality and settlement can be further understood through symbiotic analogy. For example, the symbionts of weevils have functional relatives in other insects that do not even dwell in the gut, as we see in the sawtoothed grain beetle (Hirota et al. 2017)—a pattern that may be enhanced with heightened surveying. Moreover, nutrient-provisioning symbiotic bacteria are represented by taxa that colonize both the body cavity and gut, as seen in the *Sodalis* of tsetse flies (*Glossina* spp.) (Wang et al. 2013), suggesting an ‘intermediate’ form between these nutritional symbionts. Through continued analysis of these relationships and a steady attempt to contextualize symbiont services and host biology and phylogeny, we can get closer to grasping the dynamics of these ubiquitous relationships.

Future directions in this field should include extensive sampling of host taxa for bacteria, with a greater emphasis on community partitioning based on gut morphology and physiology. Moreover, such sampling should take into account all life stages of the host. Systematic manipulation of the gut community and isolation of core community members (if present) for more thorough experimentation relating to various host services should follow these approaches. Additionally, we must further consider the community interactions between bacteria as well as other gut-dwelling organisms, including fungi, to identify symbioses between these organisms that may in turn benefit the holometabolan host. The indistinct dichotomy of facultative and obligate symbiotic bacteria in Holometabola demonstrates vague patterns than we can render clearer with additions to methodology.
